# Treatment Strategies to Prevent Renal Damage in Hypertensive Children

**DOI:** 10.1007/s11906-014-0423-2

**Published:** 2014-02-14

**Authors:** Piotr Czarniak, Aleksandra Zurowska

**Affiliations:** Department Pediatrics, Nephrology & Hypertension, Medical University Gdansk, Ul. Debinki 7, 80-211 Gdansk, Poland

**Keywords:** Hypertension, Chronic kidney disease, Obesity, Treatment, Children, Adolescents

## Abstract

Hypertension secondary to chronic kidney disease prevails in earlier childhood and obesity-related primary hypertension in adolescence. Both are associated with a high risk of renal and cardiovascular morbidity. In children with chronic kidney disease, uncontrolled hypertension may accelerate progression to end-stage renal disease before adulthood is reached and increase a child’s risk of cardiac death a thousand-fold. Obesity-related hypertension is a slow and silent killer, and though early markers of renal damage are recognized during childhood, end-stage renal disease is a risk in later life. Renal damage will be a formidable multiplier of cardiovascular risk for adults in whom obesity and hypertension tracks from childhood. Management options to prevent renal damage will vary for these different target groups. This review provides a summary of the available renoprotective strategies in order to aid physicians involved in the care of this challenging group of children.

## Introduction

Kidney damage is the second-most common complication of primary hypertension after cardiovascular events, and significantly contributes to the increasing number of adults with end-stage renal disease (ESRD) [1]. In patients with established chronic kidney disease (CKD), hypertension is one of the most important predictors of further disease progression. In both primary and secondary hypertension, renal damage represents a formidable multiplier of the patient’s global cardiovascular (CV) risk [2]. Evidence for the above statements derives from both experimental studies and a multitude of clinical studies on end-organ damage in the adult hypertensive population for whom primary hypertension is a major issue and diabetes a major cause of CKD. Most of the existing strategies to prevent renal damage and cardiovascular events in primary hypertension and CKD patients are also based on studies from adult populations. In childhood, the causes and epidemiology of both hypertension and chronic kidney disease differ from those observed in later life.

In younger children, hypertension secondary to renal disease prevails, with primary hypertension becoming a major issue in adolescence [3, 4••, 5]. The main contributor to childhood CKD is congenital abnormalities of the kidney and urinary tract (CAKUT), whereas diabetic nephropathy is a marginal cause. While the prevalence of hypertension in children and adolescents has doubled in the last two decades, it is still much lower than that observed in adults [4••]. Studies performed in the childhood hypertensive population, therefore, are scarce and frequently require a multicenter, multinational effort to reach statistically significant numbers. Cardiovascular morbidity and mortality, an important primary outcome of adult hypertensive studies, are rare events in childhood. Nevertheless, knowledge has accumulated on both the pathophysiology of hypertension-induced kidney damage in children and the treatment strategies used to prevent it. Implementation of these strategies by physicians involved in the care of the reported 4 % hypertensive children and adolescents is crucial for adequate management.

## Target Groups for Renoprotective Strategies in Childhood Hypertension

A large proportion of children with hypertension have chronic kidney disease secondary to a multitude of different renal disorders. CAKUT constitute a major group within these, and renal hypodysplasia is the most common diagnosis. The prevalence of hypertension is notably high in hereditary glomerular, microangiopathic or cystic diseases (congenital nephrotic syndrome, Alport syndrome, atypical hemolytic-uremic syndrome, ARPKD, ADPKD) [6, 7]. Severe hypertension is also seen in acquired primary and secondary glomerulopathies (FSGS, extracapillary glomerulonephritis, lupus nephritis), or following severe acute kidney injury (hemolytic–uremic syndrome, cortical necrosis) [8, 9].

The aim of treatment in children with established chronic kidney disease is prevention of additional renal damage exerted by hypertension. The goal is to slow disease progression and delay the start of renal replacement therapy (RRT). A further, equally important aim is to decrease the well-documented burden of cardiovascular risk associated with declining renal function. Many adults actually die from CV complications before reaching ESRD [10]. Though childhood death is a rare event in CKD, cardiac death rate is a thousand-fold higher than that of the age-matched general population and the major cause of mortality in children on dialysis [11–13].

The last decade has witnessed an emerging shift in the epidemiology of childhood hypertension, parallel to the epidemic of childhood obesity. Primary hypertension has been increasingly recognized in the primary care setting and has become the leading cause of hypertension in adolescents [4••]. Similarly to patients with primary hypertension, obese children with hypertension initially have intact kidneys. Renoprotective strategies for this target group are aimed at conserving normal renal function.

## Renal Damage and Preventive Strategies in Hypertensive Children with CKD

### CKD Progression

In children with CKD, once significant renal damage has occurred, progressive impairment of renal function ensues. The rate of deterioration is partially dependent upon the underlying renal disease, and children with renal hypodysplasia demonstrate a much slower progression than those with glomerulonephritis [14]. Among the modifiable factors influencing deterioration of renal function, hypertension and proteinuria have been identified as key players. Both are independent predictors of disease progression [15, 16]. Additional factors include renal anemia, hyperparathyroidism, dyslipidemia, chronic inflammation, oxidative stress, and genetic background [17]. In children, the decline in renal function is frequently not linear, and an important decrease can be noted around puberty. Data from 1,200 children with different causes of renal disease enrolled in the Italian CKD Registry (ItalKid) has shown that the risk of reaching ESRD by the age of 20 is 68 % [18].

### Pathophysiology of Hypertension in CKD

**Fig. 1:**
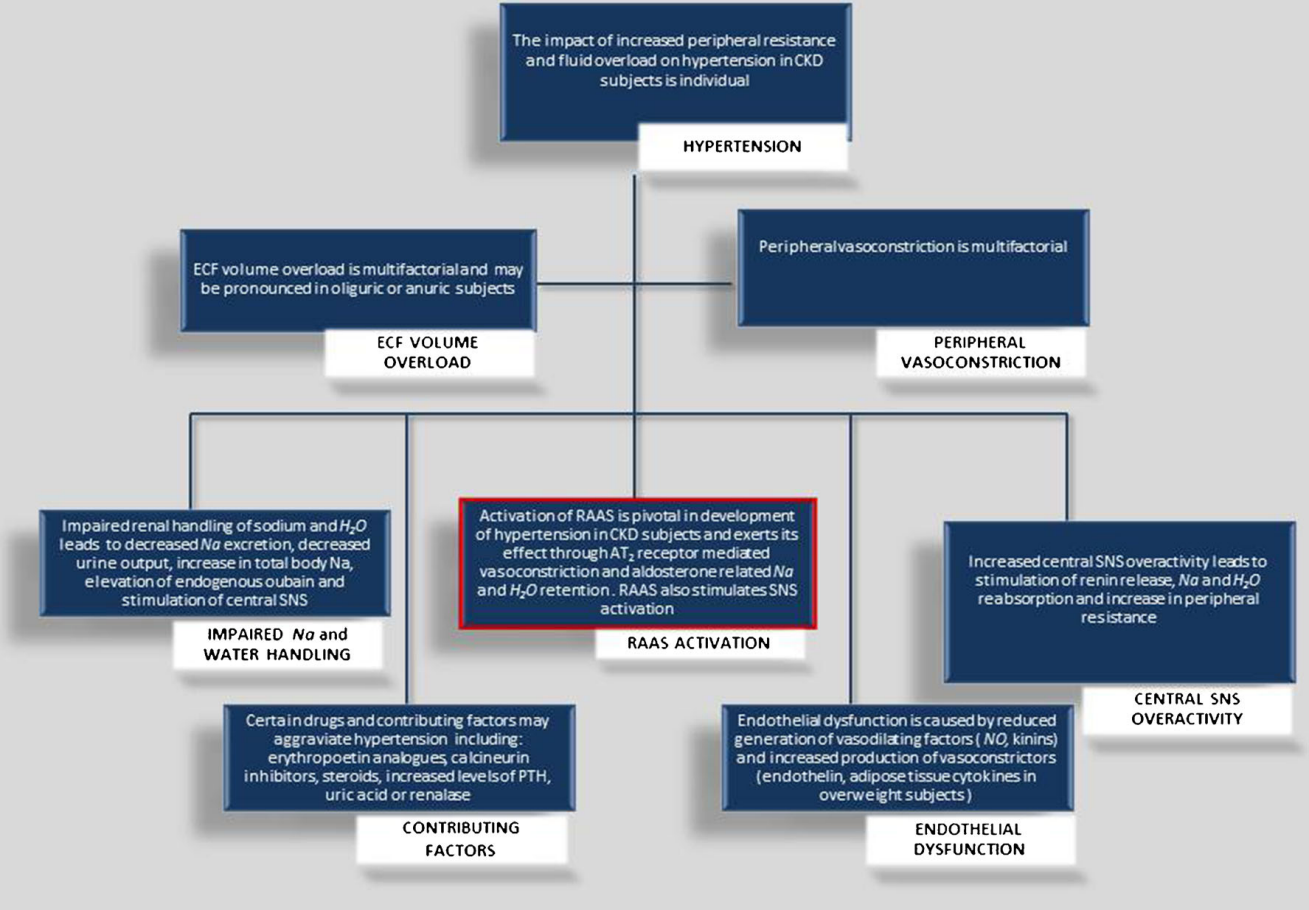
Pathophysiologic pathways of hypertension in CKD

Hypertension is highly prevalent in children with CKD, with rates ranging from 20 % to 80 %, depending upon the underlying disease and stage of renal dysfunction. The crucial pathophysiological pathways of hypertension in CKD are shown in Fig. 1. Activation of the renin-angiotensin-aldosterone system (RAAS) and fluid overload are looked upon as pivotal in the development of hypertension in CKD subjects. Angiotensin II-mediated vasoconstriction and aldosterone-mediated salt retention lead to increase in peripheral resistance and blood volume. Impaired sodium excretion leading to expansion of extracellular fluid volume and peripheral vasoconstriction is frequent in later stages of CKD [19]. Other important factors contributing to CKD hypertension are sympathetic hyperactivation, chronic hyperparathyroidism, endothelial dysfunction, reduced renalase activity, drug administration (cyclosporine, glucocorticoids, EPO) and sleep apnea [20–22].

### Therapeutic Strategies to Slow Progression of Renal Damage in CKD

The two most important strategies for slowing CKD progression that have emerged in the last years are adequate blood pressure control and minimization of proteinuria. There is less substantial data for the renoprotective effects of treating renal anemia, uremic dyslipidemia, and mineral metabolism disorders [23]. Therefore, the mainstay of renoprotective therapy in CKD is effective antihypertensive treatment, which not only attenuates worsening of renal function but also reduces proteinuria, and is effective in preventing cardiovascular events, as has been demonstrated in adults and children [12, 13, 24•].

### How Low Do We Reduce Blood Pressure in Children with CKD?

Based on initial data from large adult trials, international guidelines unanimously recommended blood pressure values below 130/80 mmHg (with some indicating lower values – 125/75 mmHg when proteinuria exceeded 1 g/day) [25]. The evidence in favor of such a low blood pressure target has been in recent dispute. Several trials have reported a paradoxical increase in mortality (so-called J-curve phenomenon) with lower blood pressure regimens [26]. Though this issue appears to be restricted to older patients with severe atherosclerosis, the results of the AASK, MDRD, and REIN-2 trials have not established with certainty that a lower BP target is justified in adults [27–29]. However, a recent meta-analysis did demonstrate a benefit of more intensive blood pressure lowering strategies on CKD progression, particularly in proteinuric patients, which was not accompanied by a detrimental cardiovascular effect [30]. This was not confirmed in non-proteinuric patients. Therefore, the target BP in CKD adults remains an area of controversy.

The results of the ESCAPE trial, an international randomized clinical study, have clearly demonstrated a beneficial effect of intensified blood pressure control on renal function in the child CKD population [31••]. In this study, 41.7 % of children in the conventional blood pressure target group (ABPM mean arterial pressure [MAP] aimed at values between 50th and 95th percentile) reached the composite endpoint of doubling serum creatinine, decline in GFR < 10 ml/min/1.73 m^2^, or start of RRT. In the intensified blood pressure target group (MAP <50th percentile), only 29.9 % of patients reached the endpoint, which corresponded to a risk reduction of 35 %. The treatment was well tolerated. European Guidelines have accordingly recommended lowering BP in children with CKD to levels below the ambulatory 75th percentile and for patients with proteinuria below the ambulatory 50th percentile [32]. In children with CKD caution in intensive blood pressure management may be necessary in individual patients with existing severe cardiovascular damage.

Blood pressure control in CKD patients exerts an additional beneficial antiproteinuric effect, as proteinuria has repeatedly been shown to be a strong independent predictor of renal progression in both adults and children [33–35]. Nevertheless nephrotic-range proteinuria was shown in a recent study of 578 children with CKD to be strongly associated with poor BP control [36]. Future research should therefore focus on strategies to reduce proteinuria, as this may improve BP control and slow the progression of CKD.

### Which Antihypertensive Agents are Best for Effective Renoprotection?

The ideal antihypertensive agent for renoprotection in children must be effective in decreasing blood pressure to target values and must target several mechanisms that induce renal injury, including RAAS activation. It must have a good pediatric safety profile and pediatric labelling, and it should need little modification with declining GFR. The various classes of available antihypertensive drugs are comparable in their ability to lower blood pressure and prevent cardiovascular events, but differ in their antiproteinuric and renoprotective effect [24•, 37]. There is strong evidence to support RAAS antagonists, such as ACE inhibitors (ACEI) and angiotensin receptor blockers (ARB), as first-choice drugs for both adults and children with CKD [25, 32, 38]. The drugs within this group block the major pathophysiological mechanism leading to hypertension in CKD (Fig. 1). In addition to their antihypertensive efficacy, they have antiproteinuric effects and an excellent safety profile. ACE inhibitors suppress the local angiotensin II tone, and ARB the angiotensin II action, whereby they reduce glomerular pressure and proteinuria, and decrease local cytokine release and activation of inflammatory pathways. This results in attenuation of glomerular hypertrophy and sclerosis, tubulointerstitial inflammation, and fibrosis. RAAS antagonists also normalize central nervous sympathetic tone and reduce oxidative stress. Randomized controlled studies have shown both their antiproteinuric efficacy and their renoprotective potential in adults with CKD [29].

In children, the ESCAPE trial demonstrated the antihypertensive and antiproteinuric efficacy of the ACE inhibitor ramipril in nearly 400 patients with CKD. However, the long-term efficacy of ACEI monotherapy was limited by a rebound of proteinuria following 24 months of treatment despite adequate blood pressure control. This so-called escape phenomenon may be due to compensatory up-regulation of ACE-independent angiotensin II production [39]. Patients with rebound proteinuria may benefit from ARB therapy or combination therapy with other drug groups. The renoprotective efficacy of RAAS antagonists has also been demonstrated in studies involving pediatric patients with CKD following hemolytic uremic syndrome and patients with Alport syndrome, conditions in which both hypertension and proteinuria are relatively severe [40, 41]. In contrast, no renoprotective effect could be demonstrated following ACE inhibition in children with hypodysplastic kidneys from the ItalKid Study [42].

Although calcium channel blockers (CCBs) achieve adequate blood pressure goals in CKD patients, the more frequently used dihydropyridine agents (amlodipine, nifedipine) have not been shown to slow progression of CKD or reduce proteinuria. Their use is limited to combination therapy with RAAS antagonists.

Beta-blockers are useful agents in reducing the overactivation of the sympathetic nervous system observed in CKD patients, resulting in reduction of cardiac output and afterload, pulse rate, and additional renal renin release. Although the renoprotective (atenolol, metoprolol) and antiproteinuric effects (metoprolol, carvedilol) of this class of drugs has been demonstrated, they are most commonly used when combination therapy is necessary for adequate blood pressure control [43–45].

Sympathetic activity has also been shown to normalize following bilateral nephrectomy, with a concomitant reduction in blood pressure [20]. A recent report demonstrated the beneficial effect of renal denervation therapy by high-frequency radio-ablation in adult patients with CKD [46]. This therapy had been previously used for the treatment of resistant hypertension in patients with preserved renal function [47].

### Combination Therapy and Dosage Regimens

Monotherapy with RAAS antagonists effectively lowers blood pressure in a subgroup of CKD children, although antiproteinuric effects may require higher doses than those registered for hypertensive treatment [48]. Recommended pediatric dosing of antihypertensive drugs and their renal clearance are available in two recent reviews on the pharmacological treatment of hypertension [49, 50]. Most ARBs (with the exception of olmesartan), CCBs, and alpha-blockers do not require dose adjustments in CKD. ACEI should be started with lower doses, and frequent response-based dose titration is necessary in advanced CKD. Beta-blockers (bisoprolol, nadolol, atenolol) that are eliminated by renal clearance require dose adjustments, while metoprolol, carvedilol, and labetalol do not, as they are metabolized by the liver.

Abnormal patterns of circadian BP rhythms in the form of nocturnal hypertension or non-dipping during sleep are common in the CKD population. This can be improved by administration of a nighttime dose of one of the prescribed antihypertensive medications [51].

When highest-approved dosage is not effective for reducing blood pressure, combination therapy is introduced. In some children, especially those with later stages of CKD, multidrug therapy is necessary. In the ESCAPE cohort, the intensive blood pressure group required an average 0.9 ± 1.1 additional drugs to high-dose ramipril (6 mg/kg/m^2^) to achieve target blood pressure [31••]. The most frequently used combination therapy for CKD patients is RAAS antagonists with a diuretic or calcium channel blocker.

The established role of the RAAS in promoting renal damage has led researchers to test whether more profound inhibition could be achieved through the concomitant use of more than one RAAS antagonist. Recent clinical trials in adult diabetic CKD patients did not confirm an additional renoprotective benefit of combined RAAS antagonist therapy [52–54]. The improvement in blood pressure and proteinuria was overshadowed by greater number of side effects, mainly hyperkalemia, emphasizing the need for a cautious and individualized approach to such treatment in the renal patient [52]. A recent editorial on the future of double-RAAS blockade in adults concluded that this therapy should be considered only for those patients in whom further cardiovascular and renal protection may be achieved without the risk of hyperkalemia or other serious side effects [55].

Diuretics are less commonly used in early stages of CKD in children. Those with hypodysplastic kidneys frequently demonstrate polyuria and may have salt-losing nephropathy. Loop diuretics are preferred and can be used also in later stages of CKD, in contrast to thiazides, which are useful only in children with GFR over 30 ml/min/1.73 m^2^. Spironolactone and the newer aldosterone antagonist eplerenone carry the same risk of hyperkalemia. Combination therapy of these agents with ARB or ACEI, therefore, is limited in CKD patients due to safety concerns.

Uric acid (UA) was recently implicated as a potential modifiable factor of CKD progression. Pilot studies suggest that lowering UA with allopurinol or febuxostat is associated with significant decrease of creatinine levels [56].

### Non-pharmacological Treatment for Hypertension

According to European guidelines, non-pharmacological treatment is an essential component in the management of every hypertensive child, including CKD subjects [32]. While the blood pressure lowering effect of weight loss, normalization of excessive sodium intake, and increased physical activity are probably the same for both non-CKD and CKD children, supporting studies are lacking. The renoprotective effect of such strategies has also not been studied. Optimal salt intake in both the general population and CKD patients is an area of controversy, although current recommendations of reducing salt intake to 5–6 g/day in adults are supported by data that higher salt intake aggravates long-term outcome [57]. Children with advanced stages of CKD and resistant hypertension require salt restriction at levels similar to the pediatric non-CKD hypertensive population. Salt restriction has been shown to increase the antihypertensive effect of RAAS inhibition.

A recent publication on the nutritional status of the pediatric renal replacement population in Europe has shown that both overweight (20.8 %) and obesity (12.5 %) are highly prevalent [58]. Therapeutic intervention to address this potentially modifiable cardiorenal risk factor is recommended for the CKD child. Exercise has been shown to decrease blood pressure in non-CKD patients. Although several studies have looked at the beneficial effect of exercise in dialysis patients, they did not address the influence of increased physical activity on lowering blood pressure or renal protection. Attenuation of renal dysfunction has been shown only in experimental animal studies [59, 60].

Sleep apnea is present in up to 30 % of the adult CKD population, particularly in those with obesity or diabetes. Improved ECF volume control with nocturnal HD, nocturnal automated PD, and nasal continuous positive airway pressure improves sleep apnea [22, 61].

## Renal Damage and Preventive Strategies in Obesity-Related Hypertension in Children

### Obesity-Related Hypertension in Children

Cross-sectional studies have found that there is a significant impact of obesity on the prevalence of childhood hypertension. A review of population studies in the United States reveals that hypertension prevalence has nearly doubled in recent decades, reaching 4 %. This has been attributed to the worldwide epidemic of obesity [4••, 62]. The long-term consequences of obesity are not only diabetes and cardiovascular damage but also renal damage [63]. Recent reviews and comments raise the concern that the ongoing obesity epidemic will be followed by a CKD epidemic, dramatically increasing the number of adults requiring renal replacement therapy in the future [64].

### Pathophysiology of Hypertension in Obesity

The pathophysiology of obesity-related hypertension involves several interacting mechanisms, including sympathetic system activation, RAAS activation, vascular endothelial dysfunction, and reduced kidney blood flow [4••, 65]. Among the recognized initiating factors are hyperinsulinemia and insulin resistance, disturbed adipocyte hormone production (increased leptin, decreased adiponectin, increased local RAAS components including angiotensinogen and aldosterone), direct compression of the kidney by perinephric fat, production of proinflammatory cytokines, oxidative stress, and sleep-disordered breathing. The complex interaction of these multiple mechanisms results in sodium and water retention, increased peripheral resistance, and impaired vasodilatory response, leading to increased blood pressure [66–68].

### Renal Damage in Obesity-Related Hypertension

The strong relationship between obesity, metabolic syndrome, and ESRD has become increasingly recognized [69, 70]. The risk of renal damage observed with increasing BMI is caused not only by hypertension and diabetes, the two most common causes of ESRD in adults, but also by metabolic syndrome [71]. Hyperinsulinemia and reduced insulin sensitivity seem to play the pivotal role, acting through multiple pathways which lead to the described obesity-related glomerulopathy characterized by glomerulomegaly and FSGS. Renal damage clinically presents with albuminuria/proteinuria and leads to progressive renal dysfunction. Onset of renal injury begins in childhood, and renal dysfunction may be recognized even before hypertension is evident. A recent pediatric study showed that mean estimated GFR values were significantly lower in obese children compared to controls [72•].

### Renoprotective Strategies for Children with Obesity-Related Hypertension

Children with obesity-related hypertension require a complex renoprotective strategy due to the multifactorial pathogenesis of kidney damage. According to current recommendations, obesity-associated hypertension requires non-pharmacological treatment including weight loss, lifestyle modifications, and salt restriction [32, 73]. This is easier said than done, and to be effective, will need to involve not only the family and medical community but also the food industry and politicians. The cornerstone of treatment is weight loss. Effective interventions to achieve and maintain weight reduction require a multidisciplinary approach to change family eating behaviors and increase family and child activity [74]. Recently debated strategies include public policy interventions aimed at improving nutritional quality of food, banning the marketing of unhealthy food, taxing sweetened drinks, and reducing the proximity of fast-food outlets to schools [75, 76].

Dietary counseling is essential, and the DASH (Dietary Approach to Stop Hypertension) diet utilized in the United States may also be effective for adolescents [77]. Lowering salt intake to recommended daily values is important due to the disturbed salt balance observed in overweight children. The benefit of salt reduction on lowering blood pressure may be more pronounced in the obese [78]. Dietary intervention includes fructose restriction, which has been associated with increased levels of uric acid, a further contributor to hypertension [79].

Current guidelines recommend 60 minutes of aerobic activity as part of a weight management strategy [74]. Physical activity may have benefits beyond BMI changes in hypertensive children. Improved blood pressure and stabilization of early markers of cardiovascular damage (cIMT, arterial stiffness), independent of BMI changes, were demonstrated in an exercise interventional trial in obese adolescents [80]. An extensive review on the treatment strategies of obesity-related hypertension was presented in an earlier issue of this journal [81].

### Pharmacological Therapy of Hypertension and Obesity

According to Endocrine Society guidelines, pharmacological therapy for obesity is considered if intensive lifestyle modifications fail to decrease weight or improve comorbidities [74]. The two most common agents are orlistat and metformin. Metformin has been shown to cause a decrease in BMI and improve markers of insulin resistance after 6 months of treatment [82]. However, the most dramatic weight reductions are reported following bariatric surgery. The International Pediatric Endosurgery Group (IPEG) has published recommendations for bariatric procedures in children and adolescents [83]. Long-term consequences of surgical treatment performed in adolescence are unknown, and longitudinal studies await finalization. To avoid nutritional deficiencies, patients must take supplements for life [84]. Nonetheless, following bariatric surgery, the resolution rates of hypertension ranged from 50--100 % [85].

Pharmacological treatment of hypertension in obese children is indicated for severe stage 2 hypertension or in the presence of diabetes or target-organ damage. Therapy may also be necessary for those that do not respond to intensive non-pharmacological management. European treatment guidelines recommend lowering blood pressure below the 90th percentile for all hypertensive obese subjects [32]. American guidelines recommend a target blood pressure below the 95th percentile and lower values (<90th percentile) for those with diabetes or target-organ damage [25].

ACE inhibitors have become the drug of choice for treating obesity-related hypertension in adults. In addition, both ACEIs and ARBs improve glucose tolerance, insulin levels, and visceral fat content, and reduce the risk of type 2 diabetes mellitus [86, 87]. While there are no available pediatric studies to support their superiority over other drug classes, ACE inhibitors seem to be the most frequently prescribed agents for children with hypertension [88, 89]. The rationale for their use in hypertensive children and adolescents is that they target the activated RAAS in patients with obesity and have an additional renoprotective effect. Evidence from diabetic and CKD populations supports the preferential choice of RAAS blockade for patients with proteinuria. Although beta-blockers target the activated sympathetic system in obese children and diuretics increase sodium excretion, they are looked upon as second-line agents due to their metabolic side effects. There has been renewed interest of late in the use of aldosterone blockade. Spironolactone and eplerenone have been shown to be effective in drug-resistant adults [90, 91].

Antihypertensive non-pharmacological interventions and pharmacological treatment will have a renoprotective effect only if blood pressure targets are lowered to the levels described above. Home and office blood pressure measurements are necessary to document the efficacy of treatment. When monotherapy is ineffective, additional drugs should be introduced. Adherence to salt reduction and other non-pharmacological strategies must be controlled in the case of resistant hypertension.

## Conclusion

Renal damage is a recognized complication of hypertension in children. The two major pediatric groups at risk are those with existing chronic renal disease and obese patients. In children with CKD, renoprotective strategy is aimed at slowing the decline of renal function in order to postpone renal replacement therapy. The mainstay of treatment is intensive blood pressure control and minimization of proteinuria. Mean arterial blood pressure is targeted below the ambulatory 50th percentile values and proteinuria below 300 mg/m^2^/day. RAAS antagonists are the first-line treatment for both hypertension and proteinuria, though many children will require multiple-drug therapy to achieve set goals.

Renoprotection in children with obesity-related hypertension is aimed at conserving normal renal function. This can be achieved by reversing the metabolic consequences of obesity through weight loss. The mainstay of treatment is non-pharmacological measures modifying lifestyle and dietary habits, increasing physical activity, and reducing salt intake. There is a limited role for effective pharmacological reversal of the metabolic consequences of obesity, and bariatric surgery is controversial. Pharmacological therapy of hypertension is introduced if non-pharmacological measures fail to lower blood pressure or if diabetes or end-organ damage is present. First-line pharmacological antihypertensive drugs are RAAS antagonists. A further common goal of renoprotective strategies in children with either primary or secondary hypertension is prevention of the formidable cardiovascular morbidity associated with declining renal function.
